# Whole-Exome Sequencing-Based Linkage Analysis of Multiple Myeloma (MM) and Monoclonal Gammopathy of Undetermined Significance (MGUS) Pedigrees

**DOI:** 10.3390/cancers17223611

**Published:** 2025-11-10

**Authors:** Alyssa I. Clay-Gilmour, Nicola J. Camp, Xiaomu Wei, Angel Earle, Aaron Norman, Jason Sinnwell, Delphine Demangel, Rosalie Griffin, Charles Dumontet, James McKay, Ken Offit, Vijai Joseph, Siwei Chen, Daniel O’Brien, Vincent Rajkumar, Robert Klein, Shaji Kumar, Steve Lipkin, Celine M. Vachon

**Affiliations:** 1 Department of Quantitative Health Sciences, Mayo Clinic, Rochester, MN 55905, USA; 2 Department of Epidemiology and Biostatistics, Arnold School of Public Health, University of South Carolina, Columbia, SC 29208, USA; earlea@email.sc.edu; 3 Huntsman Cancer Institute, University of Utah, Salt Lake City, UT 84112, USA; 4 Weill Cornell College of Medicine, University of Cornell, New York, NY 10021, USA; 5 Centre de Recherche en Cancérologie de Lyon/Hospices Civils de Lyon, Université Claude Bernard, 69373 Lyon, France; 6 Department of Epidemiology, Cancer Cancer Prevention and Population Sciences Sciences Division, The University of Texas MD Anderson Cancer Center, Houston, TX 77030, USA; rgriffin@mdanderson.org; 7 Department of Lymphoma & Myeloma, Cancer Medicine Division, The University of Texas MD Anderson Cancer Center, Houston, TX 77030, USA; 8 International Agency for Research on Cancer, World Health Organization, 69366 Lyon, France; 9 Memorial Sloan Kettering Cancer Center, New York, NY 60637, USA; 10 Department of Human Genetics, The University of Chicago, Chicago, NY 60637, USA; 11 Division of Hematology, Mayo Clinic, Rochester, MN 55905, USA; 12 Icahn School of Medicine at Mount Sinai, New York, NY 10029, USA; robert.klein@mssm.edu

**Keywords:** whole-exome sequencing, linkage analysis, multiple myeloma, monoclonal gammopathy of undetermined significance, family study

## Abstract

Background/Objectives: Family history is a known risk factor for multiple myeloma (MM) and its precursor condition, monoclonal gammopathy of undetermined significance (MGUS). Previous genome-wide association studies (GWASs) have identified 35 common loci associated with MM risk and 21 associated with MGUS. The objective of this study was to identify less common and rare genetic loci predisposing to MM/MGUS through whole-exome sequencing (WES)-based linkage analysis. Methods:Multipoint linkage analysis was conducted using the Multipoint Engine for Rapid Likelihood Inference (MERLIN) with the Lander–Green algorithm on germline WES data from 79 pedigrees with 2 or more affected relatives (120 MM, 86 MGUS, and 21 unaffected). Genome-wide linkage was evaluated using 12,946 independent single-nucleotide variants (linkage disequilibrium r^2^ < 0.05). Results: Significant linkage was observed at chromosome 6q22.33–q24.2 by the non-parametric model (logarithm-of-odds (LOD) = 3.3) and suggestive linkage by the dominant parametric model (heterogeneity LOD (HLOD) = 2.5). Fourteen rare variants within this region were prioritized using family-specific partial LOD scores and in silico functional prediction tools. Nine of these variants, *REPS1*, *THEMIS*, *TAAR6*, *AHI1*, *VNN1*, *VNN3*, *MTFR2/FAM54A*, *LAMA2*, and *PHACTR2*, overlapped immune-regulatory regions in blood cell lines and were not previously identified in GWASs. Conclusions: This study demonstrates the utility of applying a linkage analysis framework to familial WES data for identifying genomic regions and candidate genes that may contribute to MM/MGUS predisposition. These findings provide new insight into the inherited risk and etiology of familial MM and MGUS.

## Simple Summary

People with relatives who have multiple myeloma or its early condition, monoclonal gammopathy of undetermined significance, face higher risk. Most research has focused on common DNA changes, but these do not explain all inherited risk. We studied 79 families with two or more affected relatives and examined their protein-coding DNA to find regions that are passed down together with disease. We found strong evidence that a stretch of chromosome 6 (q22.33–q24.2) is linked to risk. Within this region, we highlighted 14 rare variants predicted to affect gene function; nine reside in areas that regulate immune cells. This work shows that family-based DNA linkage can uncover risk regions missed by previous approaches and points to new genes and pathways that may help explain—and ultimately predict—risk for multiple myeloma and its precursor.

## 1. Introduction

Multiple myeloma (MM) is the result of a malignant transformation of plasma cells that is preceded by the presence of an asymptomatic clonal plasma cell expansion, a condition referred to as monoclonal gammopathy of undetermined significance (MGUS) [1,2,3]. Studies have reported familial clustering of MM with MGUS, other B-cell malignancies, and solid tumors, indicating a potential shared genetic predisposition [4,5,6,7,8,9,10,11,12,13,14,15,16,17,18,19,20,21,22,23]. Studies focused on family history of MM have shown a 2- to 4-fold increased risk of MM among individuals with an affected first-degree relative [4,11,14,24]. We [22] and others [15] have shown a 2- to 3-fold increased risk of MGUS among first-degree relatives of individuals with MM or MGUS.

Genome-wide association studies (GWASs) conducted in populations of European ancestry (EA) have established 35 loci contributing to germline MM susceptibility [25,26,27,28,29,30], and 21 risk loci have been identified for MGUS [9,31,32]. Most of these variants identified are common (minor allele frequency > 0.05), confer relatively small increments in risk (odds ratios (ORs) = 1.10 to 1.63), and explain only a portion of the heritability [29,30,33]. Like other complex traits, identifying the missing heritability remains a priority [34,35].

Less common and rare variants may explain some of the missing heritability for complex traits [36,37,38]. Historically, family pedigrees have been used for identifying highly penetrant rare variants that contribute to diseases, including cancers [39,40,41,42,43,44,45,46]. Using linkage analysis, investigators can agnostically interrogate the genome for germline regions that co-segregate with disease among family members and count recombinations to delineate disease-harboring regions. Several sequencing studies, some within families, have already suggested rare variants contributing to MM susceptibility in genes, including *KIF18A* [47], *USP45/ARID1A* [48], *CDKN2A* [49], *DIS3* [50], *LSD1* [51], *BTNL2* [52], *EOMES* [52], *TNFRSF13B* [52], *IRF8* [52], *ACOXL* [52] and *TSPAN32* [52]. Catalano et al. identified 109 rare germline MM risk variants in 21 families with 46 affected and 20 unaffected members using an in-house Familial Cancer Variant Prioritization Pipeline [53]. Several of these 109 risk variants are functionally related to previously identified MM rare variants (*KMT2A/LSD1, USP28*) [53]. Niazi et al. prioritized and characterized 150 variants located in upstream, 5′ untranslated region (UTR), and 3′ UTRs from 14 MM families, reaffirming biological pathways previously implicated in MM development [54]. Given the evidence to date, a polygenic model of common and rare variants likely contributes to MM/MGUS risk.

A major challenge for complex traits is mapping potential risk variants located in regulatory regions to coding regions in the genome [55,56]. Localizing chromosomal regions using linkage analysis to target the variants is an instrument used to map traits to putative candidate genes in the genome [48]. In this study, we conduct a genome-wide linkage analysis of MM/MGUS pedigrees to identify and characterize genomic regions for MM/MGUS.

## 2. Materials and Methods

Study Population

Inclusion criteria

MM/MGUS families were recruited and ascertained from four sites: Mayo Clinic, University of Utah/Huntsman Cancer Institute, International Agency for Research on Cancer (IARC), and Memorial Sloan Kettering Cancer Center. All studies were conducted in accordance and approved by an Institutional Review Board at the respective institution. Informed written consent was obtained from all subjects. Families had at least two relatives diagnosed with MM and/or MGUS, and most consisted of a proband with confirmed MM diagnosis, meeting revised criteria of the International Myeloma Working Group. Early-onset familial MM cases were defined as those <50 years old at time of diagnosis. Unrelated, or sporadic, MM cases and controls with whole-exome data were identified from dbGaP (phs000748, phs000348, phs000179, phs000276, phs000403, phs000687, and phs000806) and used for follow-up and interrogation of linkage regions.

Whole-exome sequencing

Whole-exome sequencing (WES) data were generated from germline DNA extracted from peripheral blood from the family members. Whole-exome capture was performed using Agilent SureSelect 38 Mb(version 4) paired-end sequencing and processed on Illumina HiSeq 2000/2500 platforms (Illumina, Inc.; San Diego, CA, USA); standard alignment to GRCh37 and quality control procedures were applied. Briefly, variant calling was performed using the Genome Analysis Toolkit (GATK) HaplotypeCaller [57] in per-sample mode, followed by joint genotyping. Quality control excluded variants with <75% call rate, <8× coverage, or minor allele frequency < 0.01 (based on 1000 Genomes). Whole-exome sequencing (WES) data from all studies, including publicly available datasets, were jointly processed. Samples and variants failing QC due to sex or relationship discrepancies, low coverage, or poor quality metrics were excluded.

Linkage Analysis

**Figure 1:**
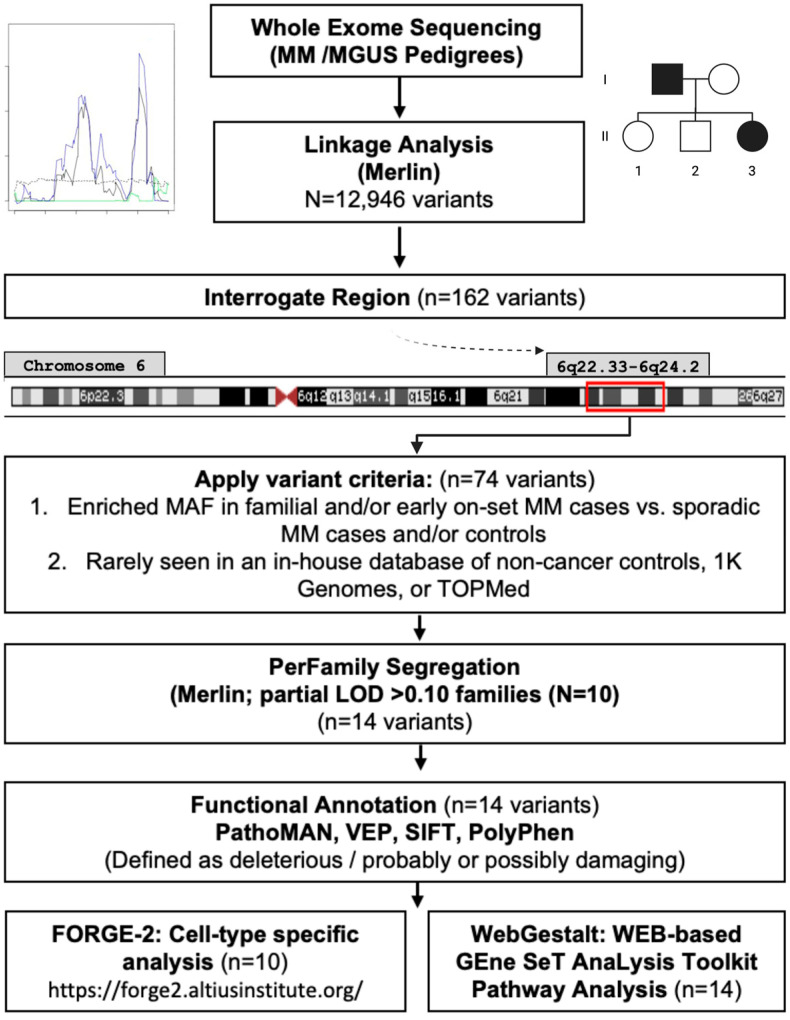
Study diagram: Linkage analysis, prioritization pipeline, and functional annotation.

Study design, workflow, and prioritization scheme are shown in Figure 1. We conducted a whole-exome sequencing (WES)-based linkage study using multipoint linkage analysis using MERLIN (Multipoint Engine for Rapid Likelihood Inference using the Lander–Green approach) [58] and evaluated both non-parametric (non-parametric linkage (NPL) analysis [59]/Kong and Cox logarithm of the odds (LOD) score [60]) and parametric (mode of inheritance: dominant/recessive-heterogeneity logarithm of the odds (HLOD) scores [61]) models to test for co-segregation of chromosomal regions with MM/MGUS. This approach leverages family-based genetic information to identify shared rare variants that may not be detectable through case–control genome-wide association studies, providing complementary insights into inherited disease risk. Pedigrees were pruned using Kinship2 v1.9.6 [62] to exclude individuals lacking phenotype or genotype data who were not essential for defining kinship among remaining members. Models were adjusted for age and sex, and MM or MGUS diagnoses were considered as affected. For the linkage analysis, variants from the WES data were filtered for independence using both PLINK’s LD-based variant pruning (r^2^ > 0.05) [63] and MERLIN’s pairwise r^2^ marker clustering approach [58]. After quality control and LD filtering, 12,946 variants remained. LOD scores >3.3 were considered to provide significant evidence for linkage [64]. Support intervals were defined as the continuous genetic region surrounding the maximum LOD score, with LOD values no less than one unit below the peak [64,65].

After linkage analysis, support regions were defined as base-pair positions > 2 LODs on either side of a segregating linkage peak ≥ 3.0. We examined all variants in this region and retained those that had a higher minor allele frequency in familial or early-onset MM cases compared to sporadic MM cases and/or controls. Prioritization was then applied to variants that met the following criteria: (1) variant must be present in all sequenced affected MM and MGUS members (2+) in at least one family, (2) variant must be rarely or less commonly seen in an in-house database of non-cancer controls (Mayo Clinic Biobank [66]), 1K Genomes, or TOPMed, and (3) variant has to be predicted to be a functionally relevant by silico models/prediction tools (described in Functional Annotation Section below) and located in coding region. We tested for overlap of known common and rare variants previously reported within the linkage results and prioritized region/variants.

Pedigree segregation

We used the ‘perFamily’ function in Merlin to identify per family contributions to log-likelihood and LOD score of the prioritized variants within the segregating region. Family pedigrees contributing a partial contribution (pLOD) of >0.10 and at least one variant within the region were interrogated individually for segregating variants and visualized using R(version 3.4.2): Kinship2 package [62].

Functional Annotation

Several in silico tools were employed to annotate plausibly functional variants within a segregating region (Figure 1). Pathogenicity of the mutation analyzer (PathoMAN) was used to further predict the functionality of the variants [67]. Briefly, PathoMAN is an automated tool for germline variant curation from clinical sequencing data, based on guidelines from the American College of Medical Genetics [67]. PathoMAN integrates diverse genomic, protein, and disease-specific data from public sources, including the Ensembl Variant Effect Predictor (VEP) [68], Polymorphism Phenotyping (PolyPhen-2) [69], and Sorting Intolerant From Tolerant (SIFT) [70]. VEP was used to assess the impact of variants, including SNPs, insertions, deletions, copy number variants (CNVs), and structural variants, on genes, transcripts, protein sequences, and regulatory regions [68]. PolyPhen-2 and SIFT were used to predict the potential effects of amino acid substitutions on protein structure and function [69,70]. We also performed a cell-type specific analysis using Functional Element Overlap Analysis of the Results of Genome-Wide Association Study Experiments (FORGE)-2 (https://forge2.altiusinstitute.org/ (accessed on 17 September 2021)), which identifies tissue- or cell type-specific signal by analyzing sets of variants that overlap with epigenetic data peaks compared to matched background variants (obtained with similar transcription start site (TSS) distance/MAF/Genomic Control (GC) to our region) [71]. FORGE2 integrates data from DNase I hypersensitive sites (DHSs), histone mark ChIP-seq broad peaks, and hidden Markov model (HMM)-based chromatin states. It enables users to (1) catalog regulatory elements overlapping regions of interest and (2) identify enrichment of regulatory features [71]. In this study, FORGE2 analyses focused on blood cell types to explore potential regulatory elements and immune-related loci or genes overlapping our target regions. Lastly, we performed pathway analysis for the variants identified as functionally relevant using WEB-based GEne SeT AnaLysis Toolkit (WebGestalt), leveraging the overrepresentation analysis (ORA) enrichment method considering human enrichment categories in annotated KEGG pathways [72].

Further details on the analytical workflow, variant prioritization, and functional annotation are provided in the Supplemental Materials.

## 3. Results

A total of 79 pedigrees were eligible for linkage analyses: 28 consisted of only MM cases, 10 had only MGUS cases, and 41 had combined MM/MGUS cases (Table S1). Annotated pedigrees consisted of 1171 individuals, 141 MM cases, 99 MGUS, and 919 unaffected (Table S2), of which 227 individuals had jointly called WES data (120 MM (9 early onset), 86 MGUS and 21 unaffected relatives) (Table 1). Median ages of diagnosis among familial MM and MGUS cases were 63 and 66 years, respectively. MM and MGUS cases were primarily male and European American (Table 1). Among unrelated individuals, there were 1183 sporadic MM cases (63 early onset), and 6808 controls, with similar ages and sex distributions to the familial cases (Table 1).

Multipoint linkage analysis

**Figure 2:**
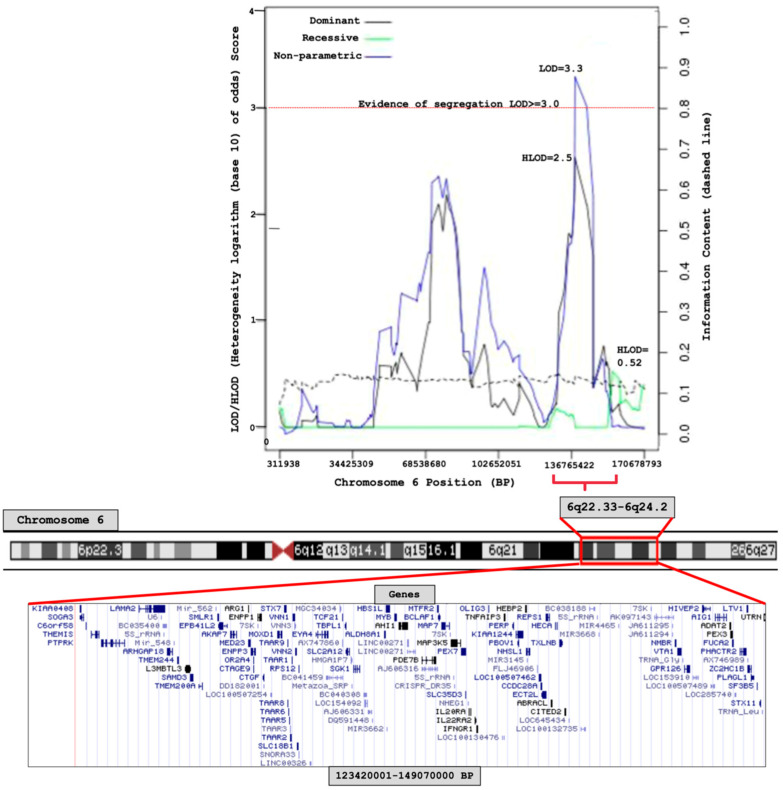
Chromosome 6 linkage (logarithm base 10 of odds) score plot of 79 MM/MGUS families.

Results from the linkage analysis are shown in Figure 2. Significant linkage was found at chromosome 6q22.33–6q24.2 (123,420,001–149,070,000, 25.6 Mb base-pair region), by the non-parametric model (LOD score = 3.3); the dominant parametric model showed suggestive evidence for linkage (HLOD = 2.5) at the same region (Figure 2). There was no evidence for linkage on any other chromosome (Figure S1).

Interrogation of region: 6q22.33–6q24.2

The 6q22.33–6q24.2 region lies outside the HLA-region and contains a total of 72 genes. We implemented the prioritization pipeline described in methods (Figure 1) and found 74 variants.

Sixteen pedigrees were identified as the strongest (partial LOD > 0.10 (range: 0.10–0.50) partial contributors to the significant linkage region; ten of those pedigrees had at least two of the seventy-four prioritized risk variants segregating within cases in a given pedigree, resulting in fourteen risk variants to further investigate (Table S3, Figure S2A–J, Table S4). These 14 variants were predicted to be functionally relevant using multiple sources (PathoMAN, VEP, Poly-Phen2, and SIFT), as either possibly/probably damaging or deleterious (Table S4). FORGE2 analysis identified regulatory elements overlapping with 9 of the 14 priority variants identified from the 6q22.33–6q24.2 region (Table S4). These nine variants (rs1044418 (*REPS1*), rs141326956 (*THEMIS*), rs17061409 (*TAAR6*), rs35851478 (*AHI1*), rs45610032 (*VNN1*), rs45623638 (*VNN3*), rs79645194 (*MTFR2/FAM54A*), rs112388307 (*LAMA2*), rs2073214 (*PHACTR2*)) were found to overlap with specific histone chromatin immunoprecipitation (ChIP) peaks, histone markers in H3K36me3-transcribed regions, and H3K27me3-polycomb-repressed regions in immune/blood cell lines (Table S4).

Pathway Analysis

Pathway analysis of the genes that contained the 14 priority variants identified significant enrichment in the Pantothenate and CoA biosynthesis (hsa00770) pathway (Enrichment Ratio = 191.34, *p* = 0.00003, FDR-*p* = 0.01) for gene set consisting of *VNN1* and *VNN3*.

## 4. Discussion

This linkage analysis identified 6q22.33–6q24.2 as a region harboring putative genes for MM and MGUS. We found several variants (n = 14) within genes (n = 12) that may contribute to this linkage signal and provide insight into the etiology of MM. While familial clustering and increased risk of disease among those with a family history of MM has been known for some time, to date, only a few family studies have utilized pedigrees to identify germline risk variants/genes for MM/MGUS.

While not all the 14 variants identified in our study have a clear link to MM/MGUS (*REPS1*, *THEMIS*, *TAAR6*, *LAMA2*, *MTFR2/FAM54A*), several (*AHI1*, *VNN1*, *VNN3*, *PHACTR2)* have had reported functional relevance in hematopoiesis and etiology of hematologic malignancies. ASNP rs2306029, in *LAMA2* (Laminin Subunit Alpha 2), has been reported [73] to increase the substantial risk for Richter syndrome, a rare transformation of chronic lymphocytic leukemia to an aggressive type. Abelson helper integration site 1 (*AHI1*) is regulated at multiple stages of hematopoiesis, with significant dysregulation observed across various human leukemic cell lines, most notably in cutaneous T-cell lymphoma (CTCL) cell lines, where *AHI1* transcript levels are elevated by up to 40-fold [74,75]. *AHI1* expression has been shown to suppress autocrine production of interleukin *(IL)-2, IL-4,* and tumor necrosis factor-alpha (TNF-α) [73]. TNF-α then induces myeloma cells to enter the cell cycle and supports the sustained growth of malignant plasma cell lines [76].

Vanin genes (*VNN1* and *VNN3*; Vascular Non-Inflammatory Molecules 1 and 3) encode members of the vanin protein family, which share high sequence similarity [77]. This family includes both secreted and membrane-associated proteins, some of which have been implicated in hematopoietic cell trafficking [77]. *VNN1* and *VNN3* exhibit pantetheinase activity, which may contribute to the oxidative stress response [78] and influence the metabolism of proteasome inhibitor-resistant MM [79]. The Pantothenate and CoA biosynthesis (hsa00770) pathway was also found to be enriched in our study. *VNN1* and *VNN3* overlap with chromatin peaks in primary peripheral blood cell lines: T helper memory, T helper/CD8+ naïve, B-cells, T-cells, neutrophils, and monocytes. They also show overlap with regulatory peaks in B cells from cord blood and hematopoietic stem cells (Table S4). The phosphatase and actin regulator 2 (*PHACTR2*) gene has been shown to be important in the response to elevated platelet cytosolic Ca2+, which has also been demonstrated as an important potential pathway in MM [80].

To further explore the biological plausibility of the prioritized genes, we examined publicly available expression data from the GTEx project (https://gtexportal.org/ (accessed on 31 October 2025)) [81]. Expression levels varied across tissues: *MTFR2* (*FAM54A*) showed high expression in EBV-transformed lymphocytes (median TPM ≈ 24), consistent with a role in B-cell-derived lineages. *REPS1* and *THEMIS* exhibited measurable expression in whole blood (median TPM ≈ 3.8 and 1.5, respectively), supporting potential immune or hematopoietic relevance. In contrast, *LAMA2* and *TAAR6* demonstrated minimal or absent expression in blood (median TPM ≈ 0.07 and 0.0), suggesting that their involvement in MM/MGUS may occur through non-hematopoietic or regulatory mechanisms rather than direct plasma-cell expression.

Several genome-wide association studies have been conducted and identified 35 common risk variants associated with MM [29]. We did not find any of these 35 common risk variants segregating in MM/MGUS families (LOD < 3.0). However, five of these known common variants, rs3132535 (*CCHCR1*; 6p21.33), rs9386514 (*ATG5*; 6q21), rs34565965 (6p22.2), rs1050976 (6p25.3), and rs74875586 (6p22.3), are located on chromosome 6, and two were found to be in strong LD (D’ = 1.0) with our identified linkage variants. These include rs3132535 (*CCHCR1*; 6p21.33), rs9386514 (*ATG5*; 6q21), rs34565965 (6p22.2), rs1050976 (6p25.3), and rs74875586 (6p22.3), which are in LD with rs150672026 (*BCLAF1*; 6q22.31). Additionally, rs74875586 (6p22.3) is in LD with rs1044418 (*REPS1*; 6q22.32). The unexpected strong LD between variants on different arms of chromosome 6 warrants further investigation to understand the genetic and structural factors contributing to this observation. Several sequencing and/or family studies have also suggested rare variants contributing to MM susceptibility in genes: *KIF18A* [47], *USP45/ARID1A* [48], *CDKN2A* [49], *DIS3* [50], *LSD1* [51], *BTNL2* [52], *EOMES* [52], *TNFRSF13B* [52], *IRF8* [52], *ACOXL* [52] and *TSPAN32* [52]. Waller et al. showed that sequencing genetically enriched MM cases, such as familial or early-onset cases, can uncover rare variants [48,52]. Using exome sequencing, they identified six recurrent, rare, and potentially deleterious variants within 5 kb of lymphoma-associated GWAS loci in 75 MM cases (*BTNL2, EOMES, TNFRSF13B, IRF8, ACOXL, TSPAN32*) [52]. All six genes replicated in an independent set of 255 early-onset, familial MM, or precursor cases. Expanded analysis of these gene regions revealed 39 rare deleterious variants, including 7 that segregated within MM families. *IRF8* (P = 1.0 × 10^−6^), *EOMES* (P = 6.0 × 10^−6^), and *BTNL2* (P = 2.1 × 10^−3^) showed significant rare variant burden in 733 sporadic MM cases versus 935 controls [52]. *BTNL2* variants at 6p21.32, while residing on the same chromosome and arm as our linkage peak, did not have significant linkage (non-parametric (NP) LOD = 0.09, *p* = 0.26). Similarly, *USP45* at 6q16 did not indicate significant linkage (NP LOD = 1.03, *p* = 0.01). Other studies have sought to utilize functional variant prioritization pipelines to identify familial MM risk variants. Catalano et al. identified 109 rare germline MM risk variants in 21 families with 46 affected and 20 unaffected members using an in-house Familial Cancer Variant Prioritization Pipeline [53]. Several of these 109 risk variants are functionally related to previously identified MM rare variants (*KMT2A/LSD1*, *USP28*) [53]. Niazi et al. analyzed 150 variants located in upstream, 5′ UTR, and 3′ UTRs across 14 MM families, ultimately identifying 20 prioritized variants [54]. None of these variants reside in our segregating region identified through linkage or indicate significant LOD scores in our families included in the analysis. This is not entirely unexpected given the rare nature of the variants and varying methods employed by each study. Together, all these studies and ours further support a polygenic model of common and rare variants likely contributing to MM/MGUS risk.

Linkage analysis is subject to several methodological and theoretical limitations that can substantially elevate the type I error rate and diminish the power to detect loci [82]. In our study, the large number of pedigrees with WES data and strict statistical significance cut-off minimize this limitation. Secondly, clinical and functional prediction tools should be interpreted cautiously as classifications can change based off new submissions and updates and can vary by the in silico model/tool being used. We used multiple up-to-date tools to characterize the variants within the segregating q22.33–q24.2 region on chromosome 6. Exome sequencing may miss regulatory intergenic regions, as well as variations that are captured at low coverage.

African Americans are about twice as likely to develop MM as European Americans (EA), and our study consists of primarily EA pedigrees, with only a small number of African American families included (7% of MM and 3% of MGUS cases). Due to limited sample size, we were unable to perform ancestry-specific analyses, and the findings likely reflect risk patterns most relevant to EA families. Future family-based studies in African American and other underrepresented populations are essential to determine whether similar or distinct germline risk variants contribute to MM/MGUS susceptibility.

Lastly, we considered MGUS and MM as one phenotype in these analyses. Even though MGUS is a necessary event prior to MM [83], their co-heritability is ~50%, and there are likely specific genetic mechanisms to the initiation of MGUS verse progression of MGUS to MM [9]. MM and MGUS were analyzed jointly due to limited sample size and because most pedigrees included a mixture of MM and MGUS cases, making phenotype-specific analyses underpowered. This approach is also supported by the high co-heritability and shared familial aggregation of MM and MGUS. However, we acknowledge that some genetic variants may differentially influence MGUS initiation versus MM progression. The significant linkage observed at 6q22.33–6q24.2 may reflect shared inherited risk across both conditions. Future studies with larger, phenotype-specific cohorts will be essential to disentangle loci contributing to MGUS initiation from those driving progression to MM.

Although the variants identified in this study are not immediately clinically actionable, they provide important biological insights into the inherited component of MM and MGUS risk. The linkage region on 6q22.33–q24.2 and the implicated immune-regulatory genes (e.g., *VNN1*, *VNN3*, *AHI1*, *PHACTR2*) highlight pathways involved in immune regulation and oxidative stress response that could represent future therapeutic or predictive targets. While direct assessment of neo-antigen potential was beyond the scope of this study, several of these genes participate in immune-related pathways that may influence tumor immunogenicity. Future studies integrating exome and transcriptomic data with HLA binding and peptide immunogenicity analyses will be essential to determine whether these variants contribute to immune recognition or host response. As functional validation and replication across diverse populations proceed, these findings may contribute to refining genetic risk models and identifying individuals at elevated risk who could benefit from early monitoring or prevention strategies.

## 5. Conclusions

In conclusion, we found significant evidence for a region on chromosome 6 (6q22.33–6q24.2), linked to MM/MGUS, and identified several genes worth further investigation. This study highlights the value of using a linkage analysis framework with familial WES data to identify genomic regions potentially involved in the development of MM and MGUS.

## Figures and Tables

**Table 1:** Characteristics of MM/MGUS cases and unaffected (related and unrelated) individuals with whole-exome sequencing data available: Cases and unaffected controls from familial and sporadic populations.

	Familial (N = 227)					Sporadic (N = 7991)		
	MM	Early-Onset ^1^ MM	MGUS	Early-Onset ^1^ MGUS	Unaffected	MM	Early-Onset ^1^ MM	Controls
Count (n, %)	120 (53)	9 (7)	86 (38)	9 (10)	21 (9)	1183 (15)	63 (5)	6808 (85)
Age at diagnosis/consent								
Median years (range)	63 (39–91)	43 (39–49)	66 (36–88)	44 (36–49)	66 (35–98)	64 (27–91)	46 (27–47)	-
missing	3 (2)	-	4 (5)	-	6 (29)	547 (46)	0	-
Sex (n, %)								
Male	64 (53)	4 (44)	44 (51)	4 (44)	12 (57)	-	-	-
Female	55 (46)	5 (56)	42 (49)	5 (56)	9 (43)	-	-	-
missing	1 (<1)	-	0	-	0	-	-	-
Ethnicity (n, %)								
European American	112 (93)	6 (67)	83 (97)	9 (100)	20 (95)	-	-	-
African American	8 (7)	2 (33)	3 (3)	0	1 (5)	-	-	-
missing	0	-	0	-	0	-	-	-

^1^ Early-onset MM/MGUS cases defined as MM/MGUS case < 50 years (included in MM/MGUS case counts).

## Data Availability

An overview of the data that supports the findings of this study are available in the supporting information of this article. The full data supporting the findings of this study are not publicly available due to privacy and confidentiality concerns. Human subjects data were obtained from multiple centers with Institutional Review Board (IRB) approval at each site. The data contain sensitive, identifiable information and are subject to compliance with HIPAA (Health Insurance Portability and Accountability Act) in the United States and GDPR (General Data Protection Regulation) in European centers, as detailed in the study.

## Supplementary Materials

The following supporting information can be downloaded at: https://www.mdpi.com/article/10.3390/cancers17223611/s1, Table S1. Affected status of pedigrees in linkage analysis. Table S2. Characteristics of MM/MGUS pedigrees (n = 79 families, 1171 members). Table S3. PerFamily Segregation analysis: Families with at least 2 prioritized variants with partial LOD scores > 0.10. Table S4. Priority variants identified in the chromosome 6 q22.33-q24.2 region (n = 24) and their predicted functional/regulatory impact. Figure S1. MM/MGUS Linkage Analysis HLOD Score Plots (Chromosomes: 1–5, 7–22). Figure S2. A–J. Pedigrees with segregating variants located in linkage peak within chromosome 6q22.33-6q24.2 region.

## Abbreviations

The following abbreviations are used in this manuscript:

CNV	Copy Number Variant
CTCL	Cutaneous T-cell Lymphoma
DHS	DNase I Hypersensitive Site
EA	European Ancestry/European American
GC	Genomic Control
GDPR	General Data Protection Regulation
GATK	Genome Analysis Toolkit
GWAS	Genome-Wide Association Study
HLOD	Heterogeneity Logarithm of the Odds
HMM	Hidden Markov Model
IL	Interleukin
KEGG	Kyoto Encyclopedia of Genes and Genomes
LD	Linkage Disequilibrium
LOD	Logarithm of the Odds
MGUS	Monoclonal Gammopathy of Undetermined Significance
MM	Multiple Myeloma
NPL	Non-Parametric Linkage
OR	Odds Ratio
ORA	Over Representation Analysis
PathoMAN	Pathogenicity of Mutation Analyzer
PLINK	Whole-Genome Association Toolset
PolyPhen-2	Polymorphism Phenotyping v2
QC	Quality Control
SNP	Single-Nucleotide Polymorphism
SIFT	Sorting Intolerant From Tolerant
TNF-α	Tumor Necrosis Factor Alpha
TOPMed	Trans-Omics for Precision Medicine
TSS	Transcription Start Site
UTR	Untranslated Region
VEP	Variant Effect Predictor
WES	Whole-Exome Sequencing
WebGestalt	WEB-based Gene Set Analysis Toolkit
