# Supplementary material for: Whole-Exome Sequencing-Based Linkage Analysis of Multiple Myeloma (MM) and Monoclonal Gammopathy of Undetermined Significance (MGUS) Pedigrees
Source: Cancers (Basel). 2025 Nov 10;17(22):3611. doi: 10.3390/cancers17223611 (PMC12651453; doi:10.3390/cancers17223611)
Supplement: Supplementary file 1 [file cancers-17-03611-s001.zip › cancers-3911357-supplementary.pdf]

Supplemental Material

Interrogation of segregating regions identified by linkage analysis

After linkage analysis, significantly segregating regions were defined as base-pair positions >2 LOD/HLODs either side of a linkage peak >= 3.0. After a segregating region was identified, we conducted downstream analyses to identify genes and/or variants in identified linkage regions that may be contributing to the signal. We examined all variants in this region and retained those that had a higher minor allele frequency in familial or early-onset MM cases compared to sporadic MM cases and/or controls. Prioritization was applied to variants that met the following criteria: (1) variant must be present in all sequenced affected MM and MGUS members (2+) is in at least one family, (2) variant must be rarely or less commonly seen in an in-house database of non-cancer controls, 1K Genomes, or TOPMed, and (3) variant had to be predicted to be a functionally relevant by silico models/prediction tools (Described in **Functional Annotation Section** below) and located in coding region.

Functional Annotation

Several insilico tools were used within the analysis workflow to annotate plausibly functional variants within a segregating region (**Figure 1**). Pathogenicity of mutation analyzer (PathoMAN) was used to further predict the functionality of the variants (Ravichandran et al., 2019). Briefly, PathoMAN automates germline genomic variant curation from clinical sequencing based on American College of Medical Guidelines. PathoMAN aggregates multiple tracks of genomic, protein and disease specific information from public sources. Some of these sources include: VEP (McLaren et al., 2016), PolyPhen-2 (Adzhubei et al., 2010), and SIFT (Ng & Henikoff, 2003). VEP was used to determine the effect of our variants (SNPs, insertions, deletions, CNVs or structural variants) on genes, transcripts, and protein sequence, as well as regulatory regions (McLaren et al., 2016). PolyPhen-2 (Polymorphism Phenotyping v2) and SIFT were used to predict possible impact of an amino acid substitution on the structure and function of a human protein (Adzhubei et al., 2010; Ng & Henikoff, 2003). We also performed a cell-type specific analysis using FORGE2 (https://forge2.altiusinstitute.org/), which identifies tissue- or cell type-specific signal by analyzing sets of variants that overlap with epigenetic data peaks compared to matched background variants (obtained with similar TSS distance/MAF/GC to our region). FORGE2 incorporates data across DNase I hypersensitive sites (DHSs), histone mark chromatin immunoprecipitation (ChIP) broadpeaks, and hidden Markov model (HMM) chromatin states. FORGE2 provides a way to 1) catalogue regulatory elements overlapping our region and 2) to identify possible enrichments. In the FORGE2 analysis for this study we focused on blood cell-types to investigate possible regulating elements and potential immune loci/genes overlapping with regions of interest.

Table S1. Affected status of pedigrees in linkage analysis

Number of affected in pedigree	Total Pedigrees
**MM-only**	
2MM	12
3MM	6
4MM	5
5MM	1
**TOTAL**	**28**
**MGUS-only**	
2MGUS	6
3MGUS	3
4MGUS	1
**TOTAL**	**10**
**MM / MGUS**	
1MM/1MGUS	11
1MM/2MGUS	5
1MM/3MGUS	2
1MM/4MGUS	1
1MM/5MGUS	1
2MM/1MGUS	8
2MM/2MGUS	6
2MM/3MGUS	2
3MM/1MGUS	1
3MM/2MGUS	1
3MM/3MGUS	2
5MM/1MGUS	1
**TOTAL**	**41**
**OVERALL TOTAL**	**79**

MM=multiple myeloma, MGUS=monoclonal gammopathy of undetermined significance, N=count

Table S2. Characteristics of MM / MGUS pedigrees (n=79 families, 1171 members)					
	**MM**	**Early-Onset[1] MM**	**MGUS**	**Early-Onset[1] MGUS**	**Unaffected**
**Count (n, %)**	141 (12)	9 (6)	99 (8)	12 (12)	919 (78)
**Whole exome sequencing available** (n=227)	**120 (85)**	**9 (7)**	**86 (86)**	**9 (10)**	**21 (2)**
**Age at diagnosis / consent**					
Median years (range)	63 (39-91)	43 (39-49)	66 (34-88)	44 (34-49)	54 (18-98)
Missing	60 (43)	-	7	-	748 (81)
**Sex (n, %)**					
Male	72 (51)	4 (44)	51 (52)	5 (42)	480 (52)
Female	68 (48)	5 (56)	48 (48)	7 (58)	451 (49)
missing	1 (<1)	-	0	-	0
**Ethnicity (n, %)**					
European American	131 (93)	6 (67)	94 (95)	11 (92)	907 (99)
African American	10 (7)	2 (33)	5 (5)	1 (8)	12 (1)
missing	0	-	0	-	0

[1]Early-onset MM/MGUS cases defined as MM/MGUS case <50 years (included in MM/MGUS case counts).

Table S3. PerFamily Segregation analysis: Families with at least 2 prioritized variants with partial LOD scores >0.10			
**Family**	**Location**	**Z-Score**	**Partial LOD**
IARC: MB-0107	chr6.143806276	1.411827	0.285119
	chr6.143754938	1.411782	0.285112
	chr6.143090803	1.409589	0.284787
Mayo_475	chr6.143754938	0.730983	0.17041
	chr6.143806276	0.730619	0.17034
	chr6.143090803	0.727069	0.169655
MSKCC_5329	chr6.143806276	0.417517	0.105323
	chr6.143754938	0.417006	0.105209
	chr6.143090803	0.410169	0.103674
MSKCC_5815	chr6.143806276	0.994502	0.218464
	chr6.143754938	0.994261	0.218422
	chr6.143090803	0.992328	0.218088
MSKCC_5885	chr6.143090803	1.326964	0.272368
	chr6.143754938	1.323659	0.271864
	chr6.143806276	1.322807	0.271734
IARC: BR-0810	chr6.143806276	2.483307	0.420349
	chr6.143754938	2.483031	0.420319
	chr6.143090803	2.474185	0.419358
IARC: BN-1110	chr6.143090803	1.388824	0.281699
	chr6.143754938	1.381445	0.280597
	chr6.143806276	1.379954	0.280374
IARC: RV-1109	chr6.143806276	2.808135	0.45422
	chr6.143754938	2.806804	0.454087
	chr6.143090803	2.790338	0.452431
IARC:NA-1109	chr6.143806276	1.247387	0.260062
	chr6.143754938	1.244579	0.259621
	chr6.143090803	1.172703	0.248186
IARC:LR-0609	chr6.143090803	0.873535	0.197063
	chr6.143754938	0.823292	0.187854
	chr6.143806276	0.811918	0.185742

Table S4. Priority variants identified in the chromosome 6 q22.33-q24.2 region (n=24) and their predicted functional/regulatory impact.

Gene	SNP	CHR / BP	ALLELES (REF/ ALT)	VEP.Consequence (variant type)[1]	SIFT TERM[2]	PolyPhen TERM[3]	FORGE-2[4] Blood cell Type		
							**Variant set[5]**	**P-value[6]**	**Data Type / Cell Line[7]**
REPS1	**rs1044418**	6:139229872	C / T	missense	deleterious	benign	rs1044418 (***REPS1***), rs79645194 (*MTFR2/FAM54A*)	5.58x10-02	Chromatin state / (E123 K562 Leukemia)
							rs1044418 (***REPS1***), rs141326956 (*THEMIS*), rs2073214 (*PHACTR2*), rs35851478 (*AHI1*), rs79645194 (*MTFR2/FAM54A*)	1.77x10-02	H3K36me3 / E046 Primary Natural Killer cells from peripheral
UTRN	rs139403938	6:144869898	G / A	missense	deleterious	probably damaging	-	-	-
THEMIS	**rs141326956**	6:128150821	T / C	missense	deleterious	probably damaging	rs141326956 (***THEMIS***)	3.54E-02	Chromatin state / E039 Primary T helper naive cells from peripheral blood
								3.83E-02	Chromatin state / E047 Primary T CD8+ naive cells from peripheral blood
							rs141326956 (***THEMIS***), rs35851478 (*AHI1*), rs79645194 (*MTFR2/FAM54A*)	5.36E-02	Chromatin state / E115 Dnd41 T-Cell Leukemia
							rs1044418 (*REPS1*), rs141326956 (***THEMIS***), rs2073214 (*PHACTR2*), rs35851478 (*AHI1*), rs79645194 (*MTFR2/FAM54A*)	1.77x10-02	H3K36me3 / E046 Primary Natural Killer cells from peripheral
TMEM200A	rs149308469	6:130762082	C / T	missense	deleterious	possibly damaging	-	-	-
BCLAF1	rs149618892	6:136600949	G / A	missense	deleterious	unknown	-	-	-
BCLAF1	rs150672026	6:136599285	G / A	missense	deleterious	possibly damaging	-	-	-
TAAR6	**rs17061409**	6:132892143	C / T	missense	deleterious	probably damaging	**rs17061409 (*TAAR6*),** rs2073214 (*PHACTR2*)	3.80E-02	Chromatin state / E030 Primary neutrophils from peripheral blood
							**rs17061409 (*TAAR6*)**	8.51E-04	Chromatin state / E062 Primary mononuclear cells from peripheral blood
							rs112388307 (*LAMA2*), **rs17061409 (*TAAR6*),** rs45610032 (*VNN1*), rs45623638 (*VNN3*)	1.89E-03	Chromatin state / E040 Primary T helper memory cells from peripheral blood
							**rs17061409 (*TAAR6*),** rs45610032 (*VNN1*)	6.93E-03	Chromatin state / E038 Primary T helper naive cells from peripheral blood
								4.28E-02	Chromatin state / E032 Primary B cells from peripheral blood
							rs112388307 (*LAMA2*), **rs17061409 (*TAAR6*),**	1.23E-02	Chromatin state / E048 Primary T CD8+ memory cells from peripheral blood
								1.56E-02	Chromatin State / E045 Primary T cells effector/memory enriched from peripheral blood
								1.80E-02	Chromatin state / E033 Primary T cells from cord blood

							rs112388307 (*LAMA2*), **rs17061409 (*TAAR6*),** rs45610032 (*VNN1*)	2.33E-02	Chromatin state / E043 Primary T helper cells from peripheral blood
								1.05E-02	Chromatin state / E047 Primary T CD8+ naive cells from peripheral blood
AHI1	**rs35851478**	6:135726105	C / T	missense	deleterious	possibly damaging	rs2073214 (*PHACTR2*), **rs35851478 (*AHI1*),** rs45623638 (*VNN3*), rs79645194 (*MTFR2/FAM54A*)	4.36E-02	Chromatin state / E035 Primary hematopoietic stem cells
							rs141326956 (*THEMIS*), **rs35851478 (*AHI1*),** rs79645194 (*MTFR2/FAM54A*)	5.36E-02	Chromatin State / E115 Dnd41 T-Cell Leukemia
							rs1044418 (*REPS1*), rs141326956 (*THEMIS*), rs2073214 (*PHACTR2*), **rs35851478 (*AHI1*),** rs79645194 (*MTFR2/FAM54A*)	1.77E-02	H3K36me3 / E046 Primary Natural Killer cells from peripheral
VNN3	**rs45623638**	6:133052580	A / C	missense	deleterious	probably damaging	rs112388307 (*LAMA2*), rs17061409 (*TAAR6*), **rs45610032 (*VNN1*), rs45623638 (*VNN3*)**	1.89E-03	Chromatin state / E040 Primary T helper memory cells from peripheral blood
							rs2073214 (*PHACTR2*), rs35851478 (*AHI1*), **rs45623638 (*VNN3*),** rs79645194 (*MTFR2/FAM54A*)	4.36E-02	Chromatin state / E035 Primary hematopoietic stem cells
							rs2073214 (*PHACTR2*), **rs45623638 (*VNN3*)**	5.20E-02	Chromatin state / E029 Primary monocytes from peripheral blood
							rs17061409 (*TAAR6*), **rs45610032 (*VNN1*)**	6.93E-03	Chromatin state / E038 Primary T helper naive cells from peripheral blood
								4.28E-02	Chromatin state / E032 Primary B cells from peripheral blood
							**rs45623638 (*VNN3*)**	8.96E-03	Chromatin state / E030 Primary neutrophils from peripheral blood
								3.64E-02	Chromatin state / E031 Primary B cells from cord blood
							rs112388307 (*LAMA2*), rs17061409 (*TAAR6*), **rs45610032 (*VNN1*)**	1.80E-02	Chromatin state / E033 Primary T cells from cord blood
								2.33E-02	Chromatin state / E043 Primary T helper cells from peripheral blood
VNN1	**rs45610032**	6:133015257	C / G	missense	tolerated	possibly damaging		1.05E-02	Chromatin state / E047 Primary T CD8+ naive cells from peripheral blood
MTFR2 / FAM54A	**rs79645194**	6:136552492	C / G	missense	deleterious	benign	rs2073214 (*PHACTR2*), rs35851478 (*AHI1*), rs45623638 (*VNN3*), **rs79645194 (*MTFR2/FAM54A*)**	4.36E-02	Chromatin state / E035 Primary hematopoietic stem cells
							rs141326956 (*THEMIS*), rs35851478 (*AHI1*), **rs79645194 (*MTFR2/FAM54A*)**	5.36E-02	Chromatin state / E115 Dnd41 T-Cell Leukemia
							rs1044418 (*REPS1*), **rs79645194 (*MTFR2/FAM54A*)**	5.58E-02	Chromatin state / E123 K562 Leukemia

							rs1044418 (*REPS1*), rs141326956 (*THEMIS*), rs2073214 (*PHACTR2*), rs35851478 (*AHI1*), **rs79645194 (*MTFR2/FAM54A*)**	1.77E-02	H3K36me3 / E046 Primary Natural Killer cells from peripheral
LAMA2	rs146462599	6:129470136	G / A	missense	tolerated	possibly damaging	-	-	-
LAMA2	**rs112388307**	6:129571288	C / T	missense	deleterious	probably damaging	**rs112388307 (*LAMA2*),** rs17061409 (*TAAR6*), rs45610032 (*VNN1*), rs45623638 (*VNN3*)	1.89E-03	Chromatin state / E040 Primary T helper memory cells from peripheral blood
							**rs112388307 (*LAMA2*),** rs17061409 (*TAAR6*),	1.23E-02	Chromatin state / E048 Primary T CD8+ memory cells from peripheral blood
								1.56E-02	Chromatin state / E045 Primary T cells effector/memory enriched from peripheral blood
								5.76E-02	H3K27me3 / E033 Primary T cells from cord blood
							**rs112388307 (*LAMA2*),** rs17061409 (*TAAR6*), rs45610032 (*VNN1*)	1.80E-02	Chromatin state / E033 Primary T cells from cord blood
								2.33E-02	Chromatin state / E043 Primary T helper cells from peripheral blood
								1.05E-02	Chromatin state / E047 Primary T CD8+ naive cells from peripheral blood
PHACTR2	**rs2073214**	6:144081609	C / T	Missense	tolerated	damaging	**rs2073214 (*PHACTR2*),** rs35851478 (*AHI1*), rs45623638 (*VNN3*), rs79645194 (*MTFR2/FAM54A*)	4.36E-02	Chromatin state / E035 Primary hematopoietic stem cells
							**rs2073214 (*PHACTR2*),** rs45623638 (*VNN3*)	5.20E-02	Chromatin state / E029 Primary monocytes from peripheral blood
							rs1044418 (*REPS1*), rs141326956 (*THEMIS*), **rs2073214 (*PHACTR2*),** rs35851478 (*AHI1*), rs79645194 (*MTFR2/FAM54A*)	1.77E-02	H3K36me3 / E046 Primary Natural Killer cells from peripheral

SNP: single nucleotide polymorphism, CHR= chromosome, BP= base-pair, ALLELES (REF/ ALT)=reference/alternative, approved gene name=VEP

[1]Ensembl Variant Effect Predictor (VEP): VEP determines the effect of your variants (SNPs, insertions, deletions, CNVs or structural variants) on genes, transcripts, and protein sequence, as well as regulatory regions.

[2]SIFT (Sort Intolerant from Tolerant): predicting amino acid changes that affect protein function

[3] PolyPhen-2 (Polymorphism Phenotyping v2) predicts the possible impact of amino acid substitutions on the stability and function proteins

[4] FORGE2: Blood-cell type specific analysis of chromosome 6 q22.33-q24.2 region

[5]Variant Set based off the 24 variants identified on chromosome 6 q22.33-q24.2 region

[6]P-value: overlap with SNP background set

[7]Data: functional elements considered; chromatin states=histone mark chromatin immunoprecipitation (ChIP) peaks (Roadmap broadPeak format) from the Roadmap Epigenomics project ; The 5 Histone marks analyzed by the tool include H3K4me1, H3K4me3, H3K27me3, H3K36me3 and H3K9me3. Each of these Histone marks is known to be enriched for a specific functional genomic category (H3K4me1-enhancer elements, H3K4me3-gene promoters, H3K27me3-polycomb-repressed regions, H3K36me3-transcribed regions and H3K9me3-repressed regions).

Figure S1. MM / MGUS Linkage Analysis HLOD Score Plots (Chromosomes: 1-5,7-22)

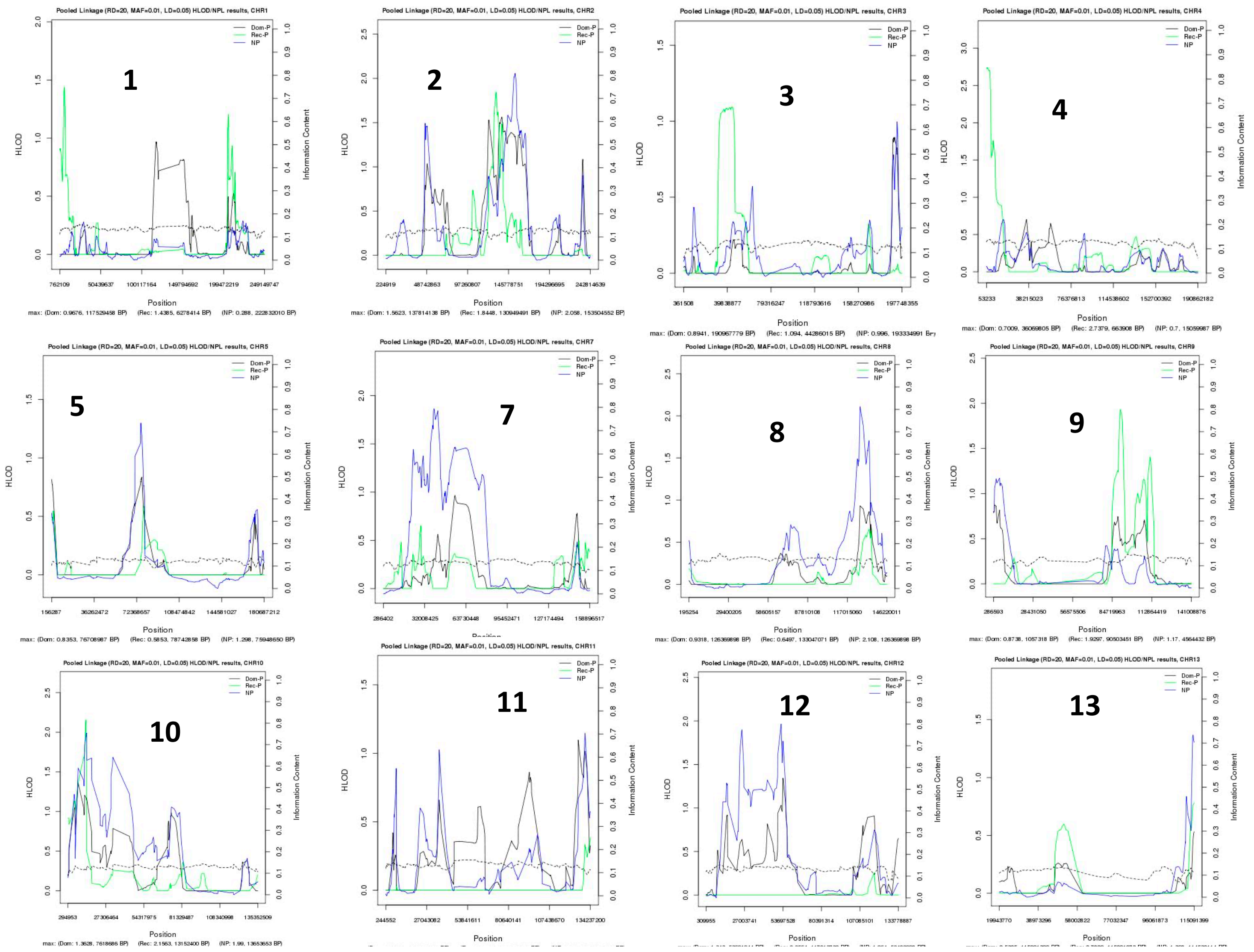

Continued: Figure S1. MM / MGUS Linkage Analysis LOD Score Plots (Chromosomes: 1-5,7-22)

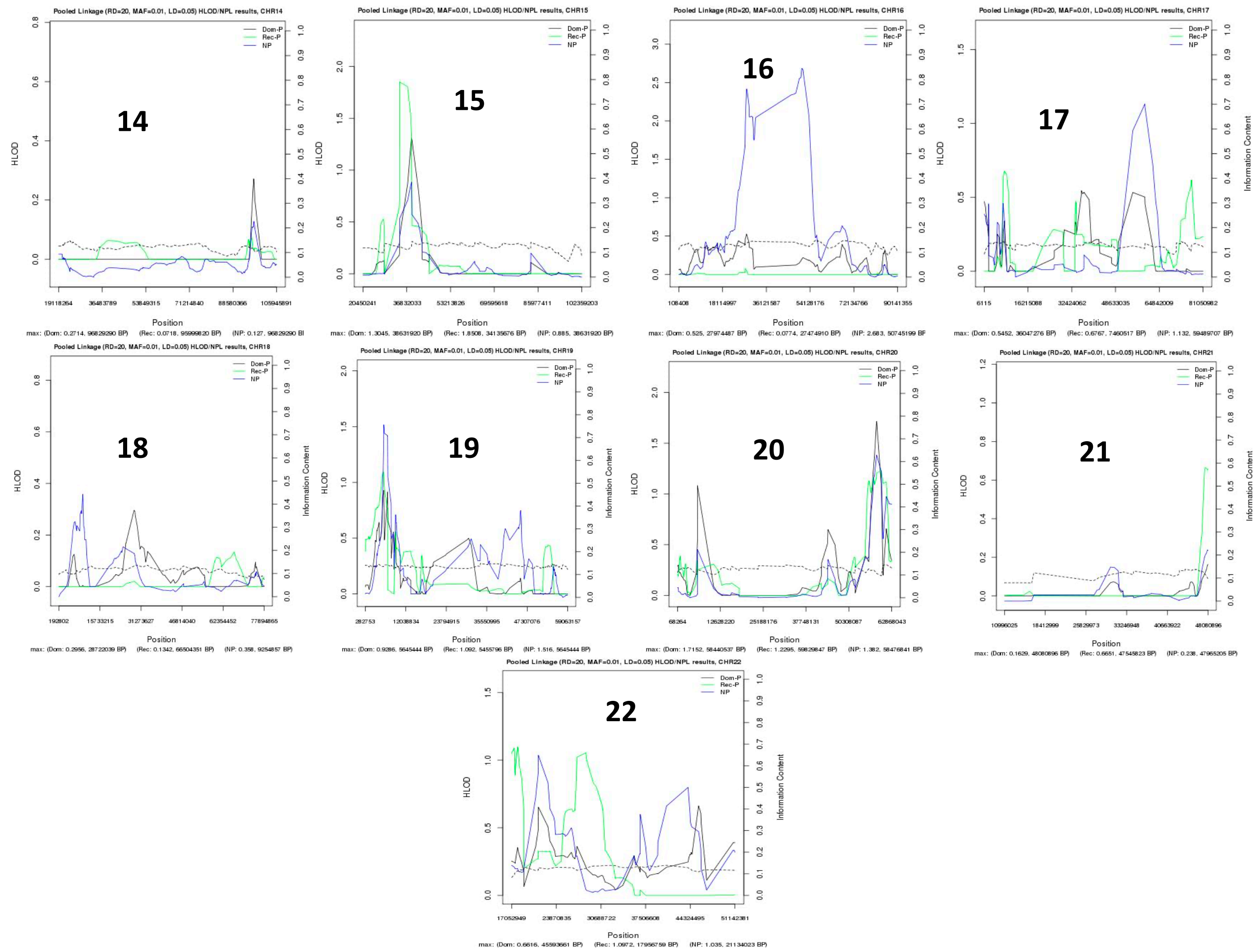

Figure S2 A-J. Pedigrees with segregating variants located in linkage peak within chromosome 6q22.33-6q24.2 region

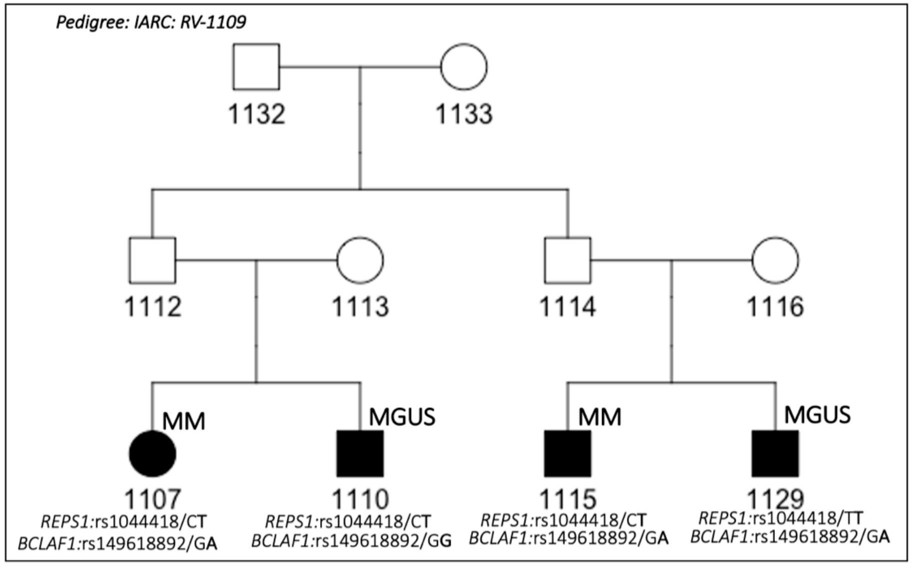

A

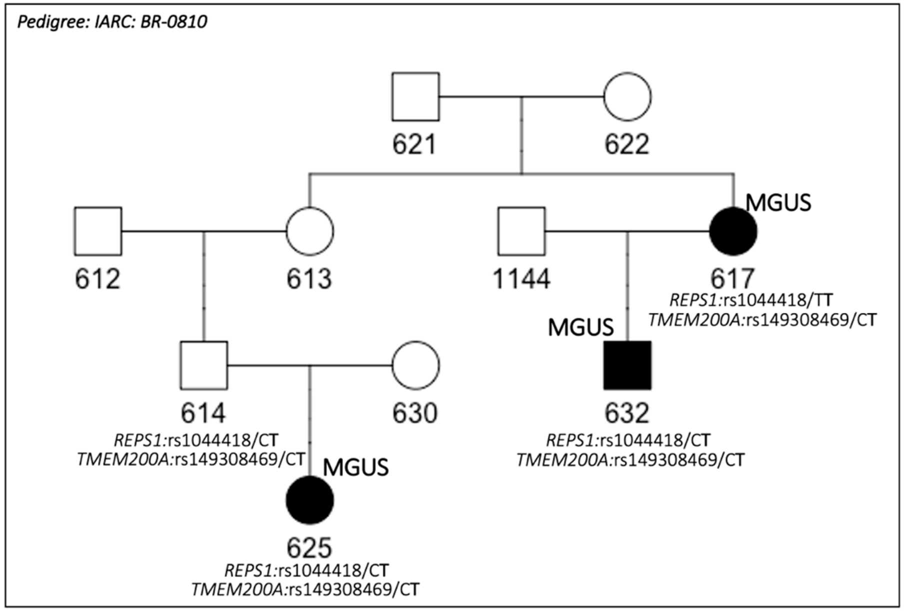
Pedigree: IARC: BR-0810
621
622
612
613
1144
MGUS
617
REPS1:rs1044418/TT
TMEM200A:rs149308469/CT
MGUS
614
630
632
REPS1:rs1044418/CT
TMEM200A:rs149308469/CT
REPS1:rs1044418/CT
TMEM200A:rs149308469/CT
MGUS
625
REPS1:rs1044418/CT
TMEM200A:rs149308469/CT

B

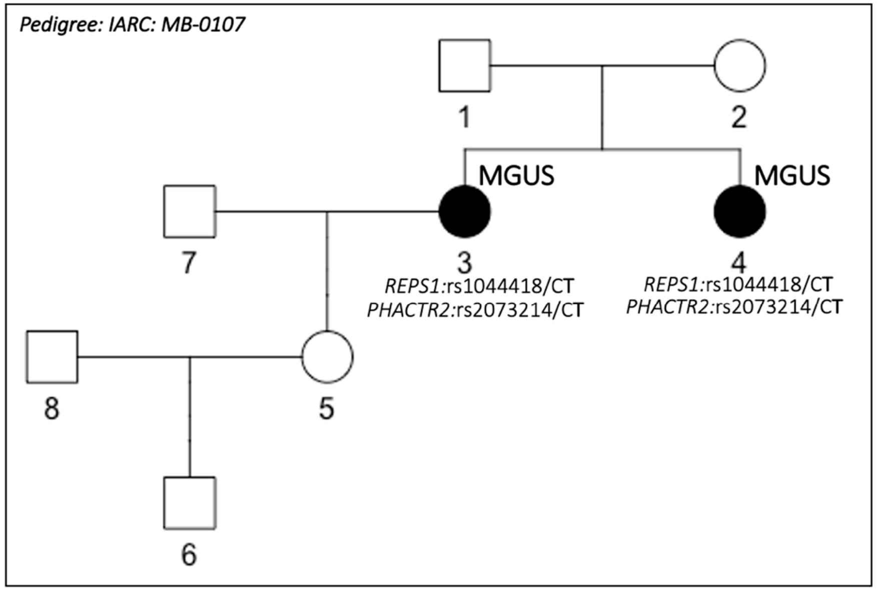
Pedigree: IARC: MB-0107
1
2
MGUS
MGUS
7
3
REPS1:rs1044418/CT
PHACTR2:rs2073214/CT
4
REPS1:rs1044418/CT
PHACTR2:rs2073214/CT
8
5
6

C

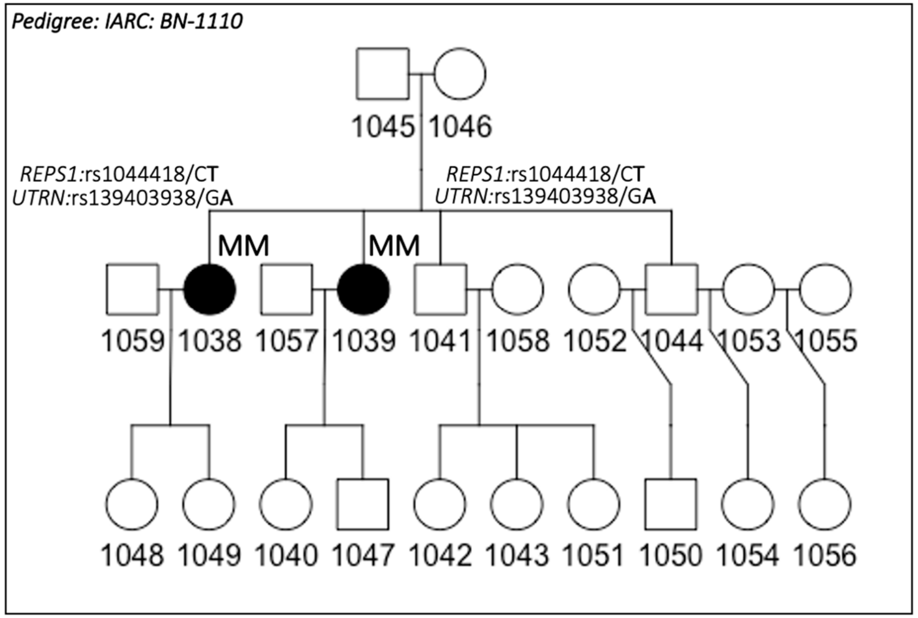
Pedigree: IARC: BN-1110
1045 1046
REPS1:rs1044418/CT
UTRN:rs139403938/GA
REPS1:rs1044418/CT
UTRN:rs139403938/GA
MM
MM
1059 1038 1057 1039 1041 1058 1052 1044 1053 1055
1048 1049 1040 1047 1042 1043 1051 1050 1054 1056

D

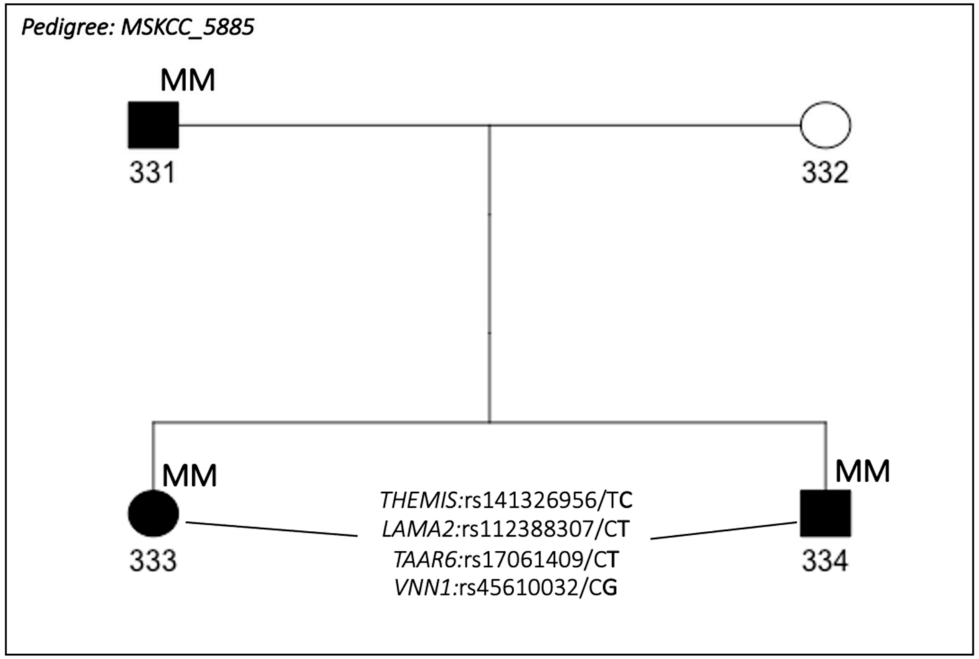
Pedigree: MSKCC_5885
MM
331
332
MM
MM
333
334
THEMIS:rs141326956/TC
LAMA2:rs112388307/CT
TAAR6:rs17061409/CT
VNN1:rs45610032/CG

E

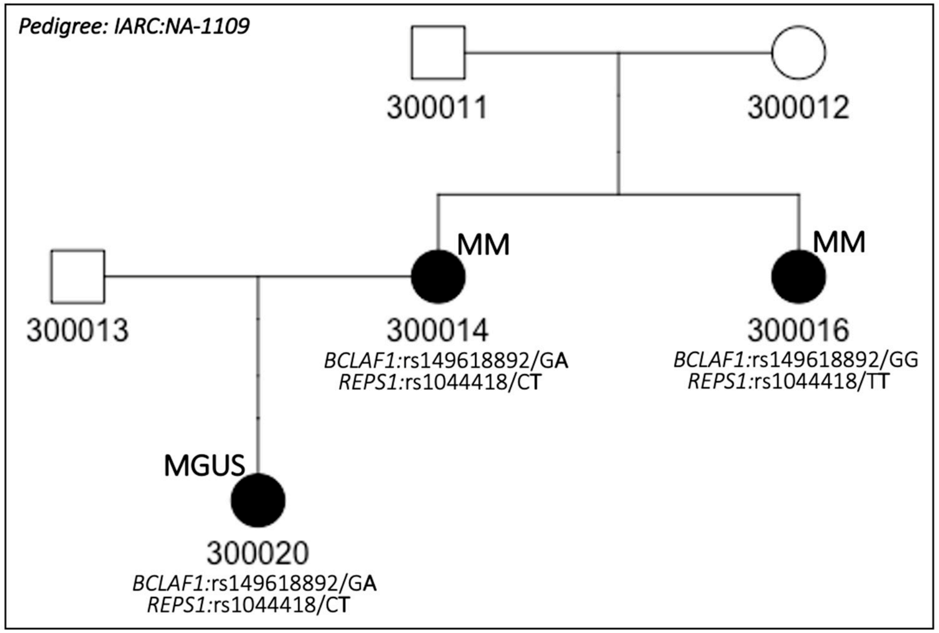
Pedigree: IARC:NA-1109
300011
300012
MM
MM
300013
300014
BCLAF1:rs149618892/GA
REPS1:rs1044418/CT
300016
BCLAF1:rs149618892/GG
REPS1:rs1044418/TT
MGUS
300020
BCLAF1:rs149618892/GA
REPS1:rs1044418/CT

F

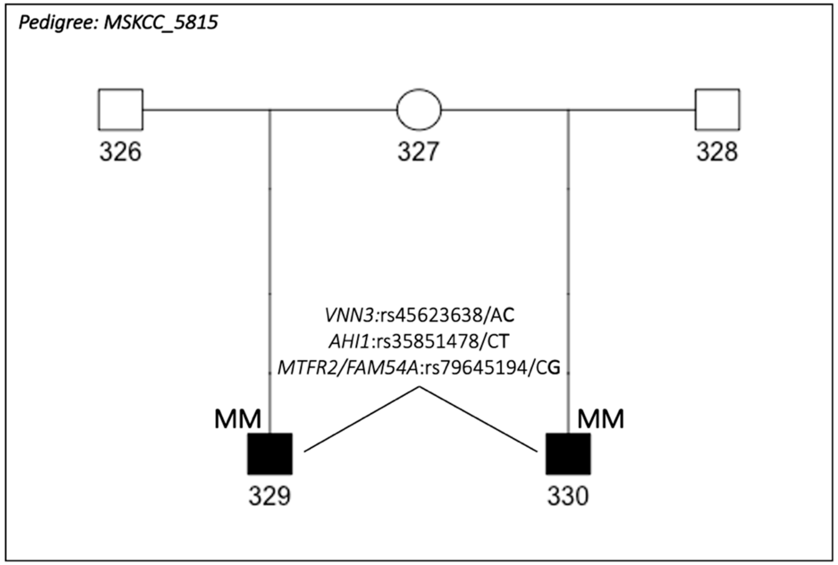
Pedigree: MSKCC_5815
326
327
328
VNN3:rs45623638/AC
AHI1:rs35851478/CT
MTFR2/FAM54A:rs79645194/CG
MM
MM
329
330

G

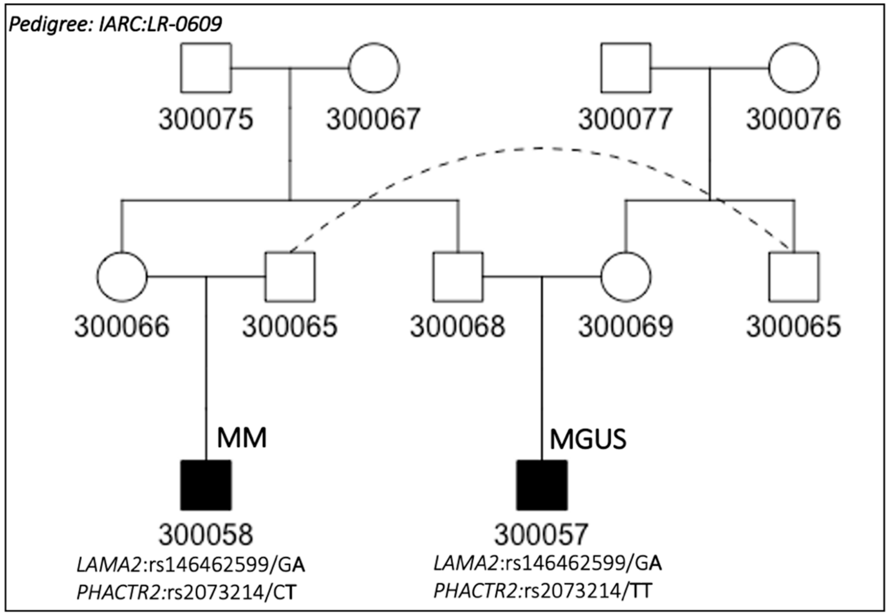
Pedigree: IARC:LR-0609
300075
300067
300077
300076
300066
300065
300068
300069
300065
MM
MGUS
300058
LAMA2:rs146462599/GA
PHACTR2:rs2073214/CT
300057
LAMA2:rs146462599/GA
PHACTR2:rs2073214/TT

H

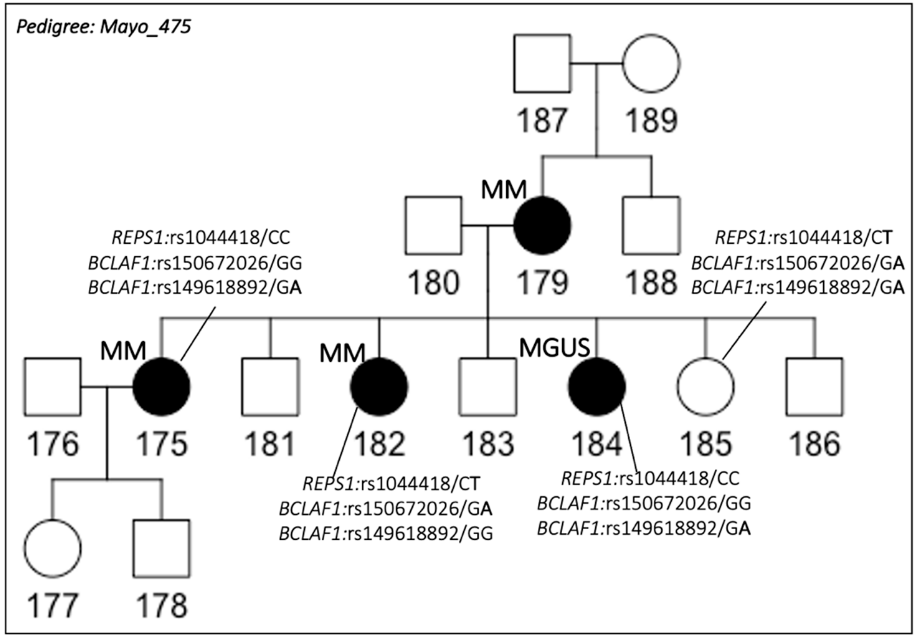
Pedigree: Mayo_475
187
189
MM
180
179
188
REPS1:rs1044418/CC
BCLAF1:rs150672026/GG
BCLAF1:rs149618892/GA
REPS1:rs1044418/CT
BCLAF1:rs150672026/GA
BCLAF1:rs149618892/GA
MM
MM
MGUS
176
175
181
182
183
184
185
186
REPS1:rs1044418/CT
BCLAF1:rs150672026/GA
BCLAF1:rs149618892/GG
REPS1:rs1044418/CC
BCLAF1:rs150672026/GG
BCLAF1:rs149618892/GA
177
178

I

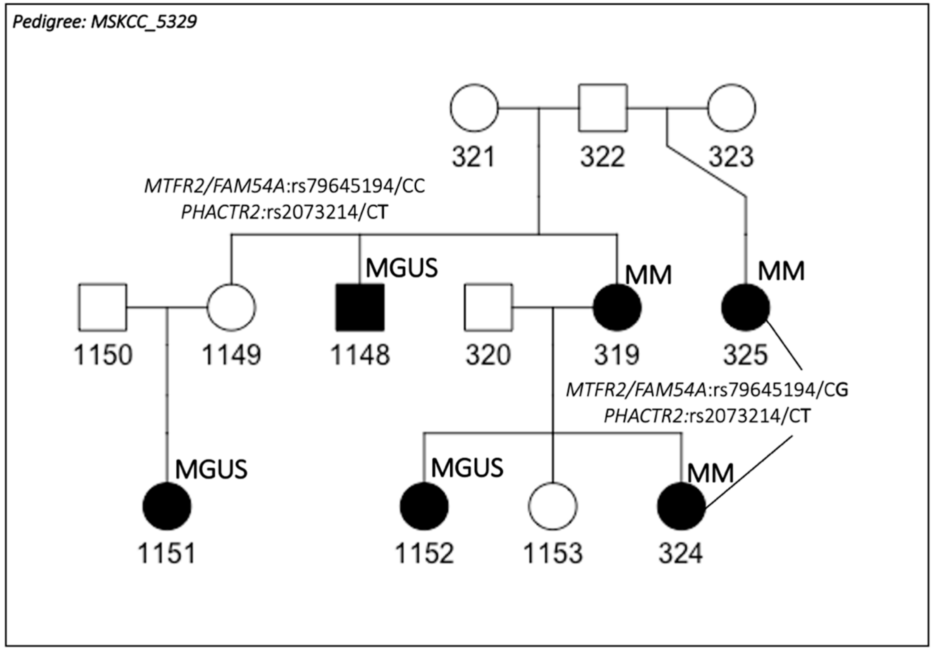

J

Adzhubei, I. A., Schmidt, S., Peshkin, L., Ramensky, V. E., Gerasimova, A., Bork, P., Kondrashov, A. S., & Sunyaev, S. R. (2010). A method and server for predicting damaging missense mutations. *Nat Methods*, *7*(4), 248–249. https://doi.org/10.1038/nmeth0410-248

McLaren, W., Gil, L., Hunt, S. E., Riat, H. S., Ritchie, G. R., Thormann, A., Flicek, P., & Cunningham, F. (2016). The Ensembl Variant Effect Predictor. *Genome Biol*, *17*(1), 122. https://doi.org/10.1186/s13059-016-0974-4

Ng, P. C., & Henikoff, S. (2003). SIFT: Predicting amino acid changes that affect protein function. *Nucleic Acids Res*, *31*(13), 3812–3814. https://doi.org/10.1093/nar/gkg509

Ravichandran, V., Shameer, Z., Kemel, Y., Walsh, M., Cadoo, K., Lipkin, S., Mandelker, D., Zhang, L., Stadler, Z., Robson, M., Offit, K., & Vijai, J. (2019). Toward automation of germline variant curation in clinical cancer genetics. *Genet Med*, *21*(9), 2116–2125. https://doi.org/10.1038/s41436-019-0463-8
